# The aPC treatment improves microcirculation in severe sepsis/septic shock syndrome

**DOI:** 10.1186/1471-2253-13-25

**Published:** 2013-09-26

**Authors:** Abele Donati, Elisa Damiani, Laura Botticelli, Erica Adrario, Maria Rita Lombrano, Roberta Domizi, Benedetto Marini, Jurgen WGE Van Teeffelen, Paola Carletti, Massimo Girardis, Paolo Pelaia, Can Ince

**Affiliations:** 1 Anesthesia and Intensive Care Unit, Department of Biomedical Science and Public Health, Università Politecnica delle Marche, via Tronto 10, 60126 Torrette di Ancona, Italy; 2 Department of Translational Physiology, Academic Medical Center, University of Amsterdam, Meibergdreef 9, Amsterdam 1105 AZ, The Netherlands; 3 Department of Physiology, Cardiovascular Research Institute Maastricht, Maastricht University, PO Box 616, 6200 MD Maastricht, The Netherlands; 4 Surgical ICU, Anesthesia and Intensive Care Department, University Hospital of Modena, Modena, Italy

## Abstract

**Background:**

The role of recombinant activated protein C (aPC) during sepsis is still controversial. It showed anti-inflammatory effect and improved the microvascular perfusion in experimental models of septic shock. The present study was aimed at testing the hypothesis that recombinant aPC therapy improves the microcirculation during severe sepsis.

**Methods:**

Prospective observational study on patients admitted in a 12-beds intensive care unit of a university hospital from July 2010 to December 2011, with severe sepsis and at least two sepsis-induced organ failures occurring within 48 hours from the onset of sepsis, who received an infusion of aPC (24 mcg/kg/h for 96 hours) (aPC group). Patients with contraindications to aPC administration were also monitored (no-aPC group).

At baseline (before starting aPC infusion, T0), after 24 hours (T1a), 48 hours (T1b), 72 hours (T1c) and 6 hours after the end of aPC infusion (T2), general clinical and hemodynamic parameters were collected and the sublingual microcirculation was evaluated with sidestream dark-field imaging. Total vessel density (TVD), perfused vessel density (PVD), De Backer score, microvascular flow index (MFIs), the proportion of perfused vessels (PPV) and the flow heterogeneity index (HI) were calculated for small vessels. The perfused boundary region (PBR) was measured as an index of glycocalyx damage. Variables were compared between time points and groups using non parametric or parametric statistical tests, as appropriate.

**Results:**

In the 13 aPC patients mean arterial pressure (MAP), base excess, lactate, PaO2/FiO2 and the Sequential Organ Failure Assessment (SOFA) score significantly improved over time, while CI and ITBVI did not change. MFIs, TVD, PVD, PPV significantly increased over time and the HI decreased (p < 0.05 in all cases), while the PBR did not change. No-aPC patients (n = 9) did not show any change in the microcirculation over time. A positive correlation was found between MFIs and MAP. TVD, PVD and De Backer score negatively correlated with norepinephrine dose, and the SOFA score negatively correlated with MFIs, TVD and PVD.

**Conclusions:**

aPC significantly improves the microcirculation in patients with severe sepsis/septic shock.

**Trial registration:**

NCT01806428

## Background

Sepsis is a major problem in intensive care units (ICU), with high incidence and mortality rate [1]. Although many studies have been conducted in order to find an effective therapy, the treatment for such a complex syndrome is still a source of investigation, mainly due to the uncertainty about its pathophysiology. According to the current hypothesis, microcirculatory alterations may contribute to the defect in oxygen extraction during sepsis and play a major role in the progression to multiorgan dysfunction [2]. For this reason the microcirculation may represent the best target for therapy aimed to improve organ dysfunction and outcome. Recombinant activated protein C (aPC) had been approved for the treatment of patients with severe sepsis in 2001, after the PROWESS Study showed that it was able to significantly reduce mortality [3]. Unfortunately, these results were not confirmed [4] and the drug was withdrawn from the market ten years later. Nevertheless, the role of aPC in sepsis therapy is still a controversial issue and papers stressing its benefits continue to be published [5-7]. APC is an endogen protein with anti-inflammatory, anticoagulant and profibrinolitic properties; it showed to inhibit the generation of thrombin by inactivating factor Va and factor VIIIa [8] and this was thought to be the most important mechanism for its therapeutic action in sepsis. Then this theory was abandoned as its anti-inflammatory action seemed to be of major relevance: preclinical studies demonstrated a host of beneficial effects targeting NF-kB pathway [9] together with the ability to reduce the apoptosis and decrease citonecrosis during sepsis [10]. Moreover aPC can influence the endothelial cell function, by inhibiting white blood cells rolling and adhesion [11], preventing the activation of inducible nitric oxide synthase (iNOS) [12] and exerting a protective role towards the endothelial glycocalyx [13]. Several experimental studies showed that aPC may integratively improve the microvascular perfusion during sepsis [14-17]. Only one study was conducted so far on severely septic patients in order to translate these experimental evidences into a clinical setting [18].

The aim of the present study was to evaluate the effect of aPC infusion on the microcirculation in patients with severe sepsis/septic shock.

## Methods

The study was approved by the Ethics Committee of AOU “Ospedali Riuniti Umberto I–Lancisi–Salesi” of Ancona (Italy) and informed consent was obtained from the patients or their relatives.

### Patient population

This prospective observational study was conducted in a 12-beds ICU of “Azienda Ospedaliera-Universitaria–Ospedali Riuniti: Umberto I–Lancisi–Salesi” of Ancona (Italy) from July 2010 to December 2011 and included patients who received an aPC infusion (24 mcg/kg/hr for 96 hrs) for the presence of severe sepsis/ septic shock and at least two sepsis-induced organ failures within 48 hours of the onset of sepsis, with no contraindications to aPC treatment (recent head trauma or intracranial bleeding). Exclusion criteria were: hematologic or advanced malignancies, liver cirrhosis, severely impaired consciousness (Glasgow Coma Scale score <7), and therapeutic limitations (do-not-resuscitate orders).

The sample size was calculated on the Microvascular Flow Index (MFI): 13 patients proved to be sufficient to demonstrate a change in MFI of 0.5 (standard deviation = 0.5) with a power of 90% and an alpha error of 0.05.

During the same study period, the microcirculation was monitored also in those patients who met the inclusion criteria but did not receive aPC because of contraindications (recent head trauma or intracranial bleeding) (no-aPC group).

### General management

All patients were sedated (propofol 2–4 mg/kg/h or midazolam 0.1–0.3 mg/kg/h and sufentanil 0.15–2 μg/kg/h or remifentanil 1.2–4.8 μg/kg/h), intubated and mechanically ventilated. All patients were equipped with a central venous catheter and a femoral artery catheter and hemodynamic parameters were monitored using the PiCCO system (Pulsion, Munich, Germany). Fluids (crystalloids and colloids), vasopressors (norepinephrine) and inotropic agents (dobutamine) were provided according to individual needs, in order to maintain the Intrathoracic Blood Volume Index (ITBVI) within the range of normality (800–1000 ml/m^2^), a normal cardiac index value, and mean arterial pressure (MAP) > 65 mmHg.

### Measurements

The Acute Physiology and Chronic Health Evaluation (APACHE) II score [19] was obtained on the day of the inclusion. Temperature (T), heart rate (HR), MAP and complete hemodynamic assessment were obtained in all patients before starting aPC infusion (T0), at 24 hours (±3 hrs) (T1a), 48 hours (±3 hrs) (T1b), 72 hours (±3 hrs) (T1c) and finally 6 hours (±3 hrs) after the end of aPC infusion (T2). Arterial blood samples were withdrawn simultaneously in order to measure blood gases, hemoglobin, and lactate levels (Omni Roche Diagnostic, Monza, Italy). Results of routine biological blood samples were collected and The Sequential Organ Failure Assessment (SOFA) score [20] was calculated daily up to day 5. Patients who did not receive aPC infusion were monitored at the same time points.

### Microcirculatory evaluation and analysis

The sublingual microcirculation was evaluated using sidestream dark field (SDF) imaging (Microscan, Microvision Medical, Amsterdam, the Netherlands) by an investigator blinded to the patient’s group at the five time points previously described (T0, T1a, T1b, T1c and T2). SDF technique is described in detail elsewhere [21]. After removal of saliva and other secretions with a gauze, the device was gently applied without pressure to the lateral side of the tongue, in an area approximately 1.5–4 cm from the tip of the tongue. Five different microcirculatory sites (at least 10 sec/site) were recorded at each time point with adequate focus and contrast and every effort was made to avoid movement and pressure artifacts. A random number was assigned to each sequence; poor-quality images were discarded and three images for each time point were selected and analyzed using a computer software package (Automated Vascular Analysis Software, Microvision Medical BV, Amsterdam, the Netherlands). According to the consensus report on the performance and evaluation of microcirculation using SDF imaging [22], Total Vessel Density (TVD), Perfused Vessel Density (PVD) and De Backer score were calculated, providing index of microvascular vessel density; the Proportion of Perfused Vessels (PPV) and the Microvascular Flow Index (MFI), reflecting microcirculatory blood flow velocity, were analyzed semiquantitatively in small- (diameter < 20 μm) (MFIs) and medium-sized vessels (20–100 μm), as described previously [23]. The Flow Heterogeneity Index (HI) was also calculated, providing an index of heterogeneous microcirculatory perfusion, which is common during sepsis [24]. Each sequence was analyzed by two investigators, both blinded to the origin of the clip: one investigator from AOU Ospedali Riuniti (Ancona, Italy) and the other from Academic Medical Center (Amsterdam, the Netherlands). Inter-observer variability was calculated for all the sequences analyzed: the coefficient of variability ranged from 4.5% to 8.7% for MFI, from 3.5% to 5.7% for the TVD and from 4.3% to 7.9% for the PPV.

### Microvascular glycocalyx assessment

SDF videos of at least 40 consecutive frames of approximately 950 μm by 700 μm sublingual tissue surface area were analyzed using the software GlycoCheck ICU (Maastricht University Medical Center, Maastricht, The Netherlands) in order to measure the Perfused Boundary Region (PBR). The PBR is the dimension of the permeable part of the endothelial glycocalyx which allows the penetration of flowing red blood cells. Erythrocytes usually have a limited access into an intact glycocalyx: when this is compromised and starts losing its protective capacity, its permeability increases, allowing circulating cells to approach the luminal endothelial membrane more closely. As a result, the dimension of the erythrocyte PBR will increase [25].

### Statistical analysis

Data were analyzed using GraphPad 5.0 program (GraphPad Software, Inc, La Jolla, CA, USA). Data are presented as median [25th–75th percentiles]. Descriptive statistics were computed for all study variables. A Kolmogorov-Smirnov test was used, and stratified distribution plots were examined to verify the normality of distribution of continuous variables. Nonparametric measures of comparison were used when variables evaluated were not normally distributed. Differences between aPC and no-aPC group were assessed using a chi-square, Fisher’s exact test, and unpaired t-test or Mann–Whitney U test as appropriate. One-way analysis of variance for repeated measures with Bonferroni post test or Friedman test with Dunn’s post-test were used to assess the evolution of microvascular perfusion in each group. Relationships between variables were assessed by Spearman’s correlation.

Differences were considered significant at (two-sided) p value < 0.05.

## Results

Thirteen patients receiving aPC were included during the study period. All the patients survived until the end of aPC infusion. General characteristics, hemodynamic and general management data are reported in Tables 1 and 2, respectively.

**Table 1:** Demographic data of aPC and no-aPC patients

		**aPC group (n = 13)**	**No-aPC group (n = 9)**	***p***
**Gender**	Male: Female	10:3	5:4	ns
**Age**	Median (min-max)	54 (31–77)	61 (34–84)	ns
**APACHE II**^**a**^	Median (min-max)	26 (16–40)	29 (20–36)	ns
**28-day mortality**	Survivors	8	5	ns
	Non-survivors	5	4	ns
**Baseline disease**	Trauma	5	3	ns
	COPD^b^	1	1	ns
	Pneumonia	2	1	ns
	Tiroidectomy	1	-	ns
	Septic shock	4	2	ns
	AAA^c^	-	1	ns
**Source of sepsis**	Lung	5	4	ns
	Urinary tract	2	1	ns
	Abdomen	3	2	ns
	Blood	3	2	ns
**Pathogen**	Gram -	9	7	ns
	Gram +	4	2	ns

^a^Acute Physiology and Chronic Health Evaluation II score.

^b^Chronic Obstructive Pulmonary Disease.

^c^Abdominal Aortic Aneurismectomy.

**Table 2:** Hemodynamic and general management data

		**Bsln**	**T1a (+24 h)**	**T1b (+48 h)**	**T1c (+72 h)**	**T2 (6 h after aPC stop)**
**MAP (mmHg)**	aPC	78 (71–86)	88 (73–95)	91 (79–94)	89 (78–98)	97 (81–102) ^#^
	no-aPC	74 (65–85)	76 (67–87)	80 (79–88)	82 (72–87)	78 (70–86)
**CI (L/min/m2)**	aPC	4.4 (3.5–5.3)	4.2 (3.5–5.4)	4.2 (3.9–4.3)	3.9 (3.4–4.7)	4.1 (3.5–4.4)
	no-aPC	4.2 (3.4–4.3)	3.9 (3–4.7)	3.9 (3.7–4.5)	4 (3.4–4.3)	4 (3.5–4.2)
**ITBVI (mL/m2)**	aPC	908 (750–1129)	878 (744–993)	863 (762–1083)	922 (799–1003)	855 (789–966)
	no-aPC	1003 (945–1135)	997 (910–1071)	970 (860–1095)	918 (853–1050)	905 (847–992)
**PaO2/FiO2**	aPC	188 (135–291)	195 (161–296)	220 (158–286)	220 (193–253)	248 (219–301) ^##^
	no-aPC	190 (189–287)	180 (173–251)	201 (184–238)	201 (156–266)	209 (162–325)
**BE (mEq/L)**	aPC	−1.9 (−7.5–1.2)	1.4 (−3.5–5.5)	3.5 (−0.3–7.2) ^#^	5.8 (−1.4–8.2) ^##^	6.3 (−0.9–8.8) ^#^
	no-aPC	2.8 (−4.8–4.9)	4.3 (0.6–6)	5.3 (−1.1–8.3)	1.7 (−2.1–8.5)	1.6 (−2.8–8.6)
**Lac (mmol/L)**	aPC	2.7 (1.5–3)	1.7 (1.4–2.8)	1.5 (1.4–2.4)	2 (1.3–2.4)	1.5 (1.1–1.9) ^# ††^
	no-aPC	2.8 (1.6–3.8)	2.3 (1.3–4.2)	1.6 (1.2–2.9)	1.2 (1–2.5)	1.5 (1.2–2.8)
**Nora (μg/kg/min)**	aPC	0.29 (0.17–0.48)	0.27 (0.16–0.48)	0.22 (0.04–0.46)	0.21 (0–0.46)	0.03 (0–0.46) ^#^
	no-aPC	0.41 (0.16–0.51)	0.41 (0.19–0.63)	0.28 (0.1–0.66)	0.25 (0.11–0.56)	0.15 (0.11–0.52)
**SOFA score**	aPC	11 (10–14)	11 (8–13)	10 (8–11)	9 (7–11) ##	8 (6–9) ### ††† **
	no-aPC	10 (8–14)	12 (7–15)	11 (7–17)	11 (7–16)	11 (9–17) ^**§**^

*p < 0.05, aPC group vs. no-aPC group at the same time point (** p < 0.01).

^#^p < 0.05, vs. T0 in the aPC-group (^##^ p < 0.01; ^###^ p < 0.0001).

^§^p < 0.05, vs. T0 in the no-aPC group.

†††p < 0.0001, vs. T1a in the aPC-group.

As shown in Table 2, MAP, PaO2/FiO2 and BE were significantly higher at T2 compared to T0, while lactate levels, norepinephrine infusion rate and SOFA score were significantly decreased.

**Figure 1:**
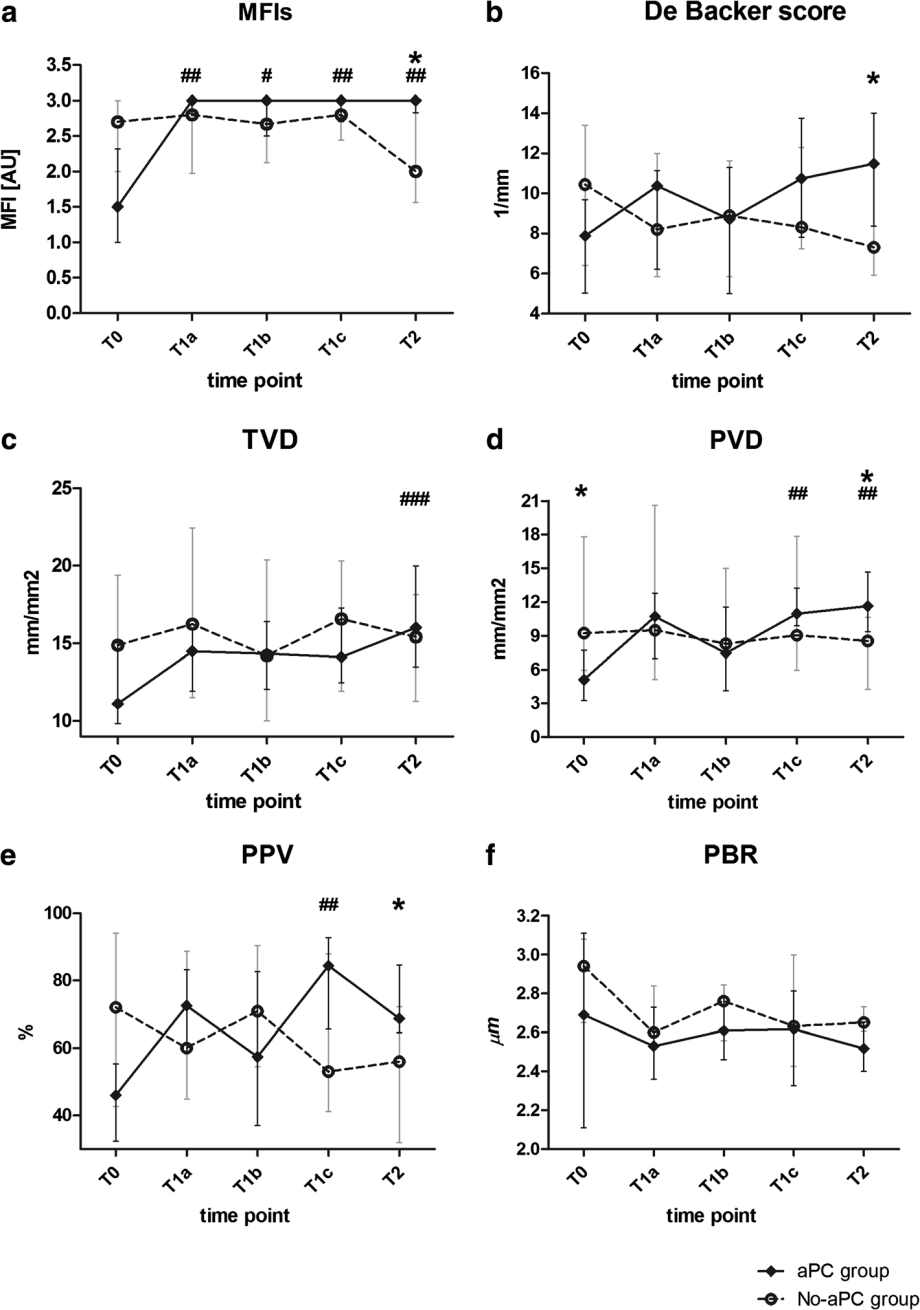
**Microcirculatory variables: aPC-group vs. no-aPC group. (a)** Microvascular Flow Index for small vessels; **(b)** De Backer score; **(c)** Total ll Vessel Density, **(d)** Perfused Vessel Density; **(e)** Proportion of Perfused Vessels; **(f)** Perfused Boundary Region. ^#^ p < 0.05, vs. T0 in the aPC-group (^##^ p < 0.01; ^###^ p < 0.0001). * p < 0.05, aPC-group vs. aPC-group at the same time point.

Microcirculatory variables are shown in Table 3. All the microvascular parameters improved over time, with significant increases in MFI, MFIs (Figure 1A), TVD (Figure 1C), PVD (Figure 1D) and PPV (Figure 1E) and a decrease in the HI. A slight not significant decrease in PBR could be noted over time (Figure 1F).

**Table 3:** Microcirculatory data

		**Bsln**	**T1a (+24 h)**	**T1b (+48 h)**	**T1c (+72 h)**	**T2 (6 h after aPC stop)**
**MFI (AU)**	aPC	1.7 (1.5–2.3)	2.9 (2.5–3) ^##^	3 (2.5–3) ^#^	2.9 (2.7–3) ^#^*	3 (2.9–3) ^###^**
	no-aPC	2.7 (1.5–3)	2.3 (2–3)	2.6 (1.7–2.9)	2.5 (2–2.6)	1.7 (1.6–2.8)
**MFIs (AU)**	aPC	1.5 (1–2.3)	3 (2.8–3) ^##^	3 (2.5–3) ^#^	3 (2.7–3) ^##^	3 (2.8–3) ^##^ *
	no-aPC	2.7 (2–3)	2.8 (2–3)	2.7 (2.1–3)	2.8 (2.4–3)	2 (1.6–3)
**TVD (mm/mm2)**	aPC	11.1 (9.8–15)	14.5 (11.9–16)	14.3 (12–16.4)	14.1 (12.5–17.3)	16 (13.5–20) ^###^
	no-aPC	14.9 (11.4–19.4)	16.2 (11.5–22.5)	14.2 (10–20.4)	16.6 (11.9–20.3)	15.4 (11.3–18.2)
**PVD (mm/mm2)**	aPC	5.1 (3.2–7.7) *	10.7 (7–12.8)	7.4 (4.1–11.6)	11 (10–13.2) ^##^	11.6 (9.4–14.7) ^##^ *
	no-aPC	9.2 (5.9–17.8)	9.5 (5.1–20.6)	8.3 (7.1–15)	9 (6–17.9)	8.5 (4.3–10.6)
**PPV (%)**	aPC	46 (32–55)	73 (59–83)	57 (37–82)	84 (66–93) ^##^	69 (65–85) *
	no-aPC	72 (43–94)	60 (45–89)	71 (54–90)	53 (41–88)	56 (32–72)
**De Backer score (n/mm)**	aPC	7.9 (5–9.7)	10.4 (6.2–11.1)	8.7 (5–11.3)	10.7 (7.8–13.8)	11.5 (8.4–14) *
	no-aPC	10.5 (6.4–13.4)	8.2 (5.8–12)	8.9 (5.8–11.6)	8.3 (7.2–12.3)	7.3 (5.9–11.3)
**HI (AU)**	aPC	0.5 (0.13–1.5)	0.25 (0–0.5)	0 (0–0.42)	0 (0–0.5)	0 (0–0) ^#^ *
	no-aPC	0.07 (0–0.41)	0.07 (0–0.57)	0.07 (0–0.58)	0 (0–0.42)	0.5 (0–1.75)
**PBR (μm)**	aPC	2.69 (2.11–3.11)	2.53 (2.36–2.73)	2.61 (2.46–2.79)	2.62 (2.33–2.81)	2.52 (2.40–2.65)
	no-aPC	2.94 (2.65–3.08)	2.60 (2.54–2.84)	2.76 (2.56–2.84)	2.63 (2.43–3.00)	2.65 (2.61–2.73)

^#^p < 0.05, vs. T0 in the aPC-group (^##^ p < 0.01; ^###^ p < 0.0001).

*p < 0.05, aPC group vs. no-aPC group at the same time point (**p < 0.01).

Nine patients with contraindications to aPC infusion were monitored. All of them survived until the end of the study protocol. No-aPC patients did not significantly differ from aPC patients for age, gender, APACHE II score (Table 1), SOFA score, hemodynamic parameters and blood gas values (Table 2). Baseline microcirculatory variables were similar in no-aPC and aPC patients, except for PVD which was significantly lower in the aPC-group (p < 0.05, Table 3). Hemodynamic, blood gases and the microvascular flow did not show any significant change during the whole study period. A significant increase in the SOFA score was seen at T2 compared to T0 (Tables 2 and 3, Figure 1).

As shown in Table 2 and Figure 1, in aPC patients compared to no-aPC the MFI was higher at T1c (p = 0.02) and T2 (p = 0.001), MFIs, PVD, PPV and De Backer score were higher (p = 0.03, p = 0.04, p = 0.04, p = 0.03 respectively) and HI was lower (p = 0.01) at T2.

A significant correlation was found between MFIs and MAP (r = 0.3, p < 0.01) but their changes over time were not correlated. TVD, De Backer score and PVD were negatively correlated with norepinephrine dose (r = −0.4, p < 0.01; r = −0.3, p < 0.05; r = −0.2, p < 0.05, respectively). No relationship was seen between changes in CI or in ITBVI and changes in microcirculatory variables. The SOFA score was negatively correlated with MFIs (r = −0.3, p < 0.01), TVD (r = −0.5, p < 0.01) and PVD (r = −0.4, p < 0.01).

## Discussion

The present study confirms that aPC treatment improves the microcirculation in severe septic/septic shock patients. This is in accordance with the conclusions of De Backer et al. [18], who studied the effects of aPC on the microcirculation using orthogonal polarization spectral imaging and evaluated changes in the De Backer score and PPV. We tried to perform a more comprehensive analysis of the microcirculation by calculating further parameters for vessel density (TVD, PVD), microvascular perfusion (MFI, PPV) and flow distribution (HI). According to De Backer’s findings, we found an improvement in the microvascular density following aPC infusion, suggesting a recruitment of capillaries which were not perfused before, and an increase in the amount of continuously perfused vessels. Additionally, the flow in the smaller vessels was improved and more homogeneously distributed–as results from the increase in MFIs and the decrease in the HI, respectively–suggesting a benefit of aPC on the rheology of the microcirculation during sepsis.

Moreover, whereas we found a stable improvement in the microcirculation, De Backer et al. had reported a transient early worsening in microcirculatory variables after aPC cessation, although they had neither indicated significant differences towards previous days, nor found a persistence of such a deterioration in the following 3 days. This discrepancy is not immediately clear to explain: indeed, aPC was infused at the same dose in both studies; differences in patient baseline characteristics between the two studied cannot be excluded, although baseline SOFA scores were similar in the two studies. Differences in time point measurements may have played a role: contrarily to De Backer, we only evaluated the microcirculation every 24 hours and might have missed a temporary slight alteration occurring during the end of aPC infusion. Still, if such a transient deterioration did occur, this might do nothing but confirm the role of aPC in influencing the microvascular function: indeed, it is logical to expect the end of the administration of a drug to be reflected by a transient instability at the level where it should act. Actually, looking at our raw data, the PPV seemed to slightly decrease after aPC cessation, even if the difference towards previous time points was not significant.

Data for this study had been collected before the publication of the PROWESS SHOCK trial and the withdrawal of aPC from the market. However, discrepancies between PROWESS and PROWESS SHOCK trials are still a matter of concern [26]. Several experimental evidences support the beneficial effect of aPC on the microcirculation [14-17]. In an experimental model of sepsis, Marechal et al. showed that aPC administration was able to preserve either microvascular perfusion and glycocalyx integrity [13]. Nevertheless, in our study the microvascular recovery seems not to be related to a glycocalyx restoration, although a slight even if not significant PBR decrease was found during aPC infusion.

A significant decrease in blood lactate and an increase in BE were seen during aPC treatment, which may reflect an improved tissue oxygen uptake. This is consistent with our previous findings: using near infrared spectroscopy, we demonstrated that aPC infusion may increase tissue oxygenation and microvascular reactivity in severely septic patients, leading to an improved cellular metabolism [27]. MAP also increased after aPC administration. Nevertheless, this does not seem to be the reason for the microvascular improvement, since either we and De Backer found no relationship between changes in MAP and the improvement in MFIs. Moreover, CI and ITBVI did not change in aPC- nor in no-aPC patients and were not correlated to microvascular variables, according to previous evidences [28,29]. We agree with De Backer in rather relating the increase in MAP to an effect of a better microvascular tone. Indeed, many experimental studies demonstrated that aPC can protect the microcirculation from endotoxic shock thanks to its anti-inflammatory properties [15,30-32] and the rise in MAP can be reasonably explained by its ability to inhibit the iNOS, thereby preventing the arteriolar vasodilation [12].

The case-crossover analysis, in which each patient served as his/her own control, is a major limitation of our study. We cannot be sure that the microvascular improvement depended on different treatments or the independent evolution in the patient’s condition rather than aPC infusion.

We monitored 9 severe sepsis/septic shock patients who did not receive aPC because of contraindications. The fact that these patients did not show any improvement in the microcirculation over time would suggest that aPC infusion can really exert a beneficial effect on microvascular perfusion. However, beyond the lack of differences in general characteristics and the collected baseline clinical data, these patients were different from aPC-patients by definition and the comparison between them cannot provide any conclusion. As a major limitation, our analysis is lacking of a real control group with patients adequately matched to those receiving aPC, which would allow to reliably discriminate the effects of aPC on the microcirculation by controlling for possible confounding factors. However, the case crossover design was required for ethical reasons.

A further limitation of our study is that many factors were not considered which might have independently affected the microcirculation. Moreover, the sample was too small and heterogeneous to adjust for potential confounders.

The ability to improve the microcirculation would be of major importance since the persistence of microvascular alterations during septic shock proved to be associated with the occurrence of organ failures and mortality [28]. In our cohort, microvascular flow and density negatively correlated with norepinephrine requirement and SOFA score. Unfortunately, our study was not designed to look at mortality. Therefore, the relation between aPC treatment and outcome could not be investigated, nor was it possible to reliably test an association between the microvascular improvement and survival. As a limitation, our data do not allow to support that an aPC-induced improvement in microvascular perfusion may eventually result in lower risk of mortality.

## Conclusions

The present study supports a role of aPC treatment in improving the microvascular perfusion in severe sepsis/septic shock patients. The relationship between the aPC-induced microvascular improvement and the outcome is still to be demonstrated.

## Abbreviations

ICU: Intensive care unit; aPC: Activated protein C; MAP: Mean arterial pressure; CI: Cardiac index; ITBVI: Intrathoracic blood volume index; TVD: Total vessel density; PVD: Perfused vessel density; MFI: Microvascular flow index; MFIs: Microvascular flow index for small vessels; PPV: Proportion of perfused vessels; HI: Flow heterogeneity index; PBR: Perfused boundary region; APACHE II: Acute physiology and chronic health evaluation II; SOFA: Sequential organ failure assessment; T: Body temperature; HR: Hearth rate; BE: Base excess; SDF: Sidestream dark field.

## Competing interests

CI is the inventor of sidestream dark field imaging technology and holds shares in MicroVision Medical and was a consultant for this company more than four years ago but has had no further contact with the company since then. He has no other competing interests in this field other than his commitment to promoting the importance of the microcirculation during patient care; and no other relationships or activities that could appear to have influenced the submitted work.

The other authors have no competing interests to declare.

## Authors’ contributions

AD and CI conceived, designed and supervised the study. LB, MRL, RD, BM, PC made substantial contribution to the acquisition of the data. LB, MRL, RD and JWGEvanT analyzed microcirculation videos. AD and ED interpreted the data, performed the statistical analysis and drafted the manuscript. GM, PP and EA supervised and evaluated the study and helped to interpret the data. All authors had full access to the data and take responsibility for the integrity of the data and the accuracy of the analysis. All authors read and approved the final manuscript.

## Pre-publication history

The pre-publication history for this paper can be accessed here:

http://www.biomedcentral.com/1471-2253/13/25/prepub
